# The effect of turbidity on recognition and generalization of predators and non-predators in aquatic ecosystems

**DOI:** 10.1002/ece3.454

**Published:** 2012-12-27

**Authors:** Douglas P Chivers, Fawaz Al-Batati, Grant E Brown, Maud C O Ferrari

**Affiliations:** 1 Department of Biology, University of SaskatchewanSK, S7N 5E2, Canada; 2 Department of Biology, Concordia UniversityQC, H4B 1R6, Canada; 3 Department of Biomedical Sciences, WCVM, University of SaskatchewanSK, S7N 5B4, Canada

**Keywords:** Anthropogenic change, anti-predator behavior, fathead minnows, predator recognition, risk assessment, turbidity

## Abstract

Recent anthropogenic activities have caused a considerable change in the turbidity of freshwater and marine ecosystems. Concomitant with such perturbations are changes in community composition. Understanding the mechanisms through which species interactions are influenced by anthropogenic change has come to the forefront of many ecological disciplines. Here, we examine how a change in the availability of visual information influences the behavior of prey fish exposed to potential predators and non-predators. When fathead minnows, *Pimephales promelas*, were conditioned to recognize predators and non-predators in clear water, they showed a highly sophisticated ability to distinguish predators from non-predators. However, when learning occurred under conditions of increased turbidity, the ability of the prey to learn and generalize recognition of predators and non-predators was severely impaired. Our work highlights that changes at the community level associated with anthropogenic perturbations may be mediated through altered trophic interactions, and highlights the need to closely examine behavioral interactions to understand how species interactions change.

## Introduction

Anthropogenic change affecting the environment is leading to a rapid alteration of global biodiversity (Vitousek 1994), and habitat alteration has been identified as one of the “Big Five” drivers of biodiversity loss (Sala et al. 2000). Identifying and understanding potential mechanisms through which species interactions are influenced by anthropogenic change has come to the forefront of many ecological disciplines, including species invasions and conservation (Kleiman 1989; Dobson et al. 1997; Skelly et al. 2007; Courchamp et al. 2008). One underappreciated way to better understand how habitat alteration affects species interactions is to look at the effect of those changes at the behavioral level. Although ecological endpoints, such as population size or growth rate, may give an overview of how populations are changing through time, they do not provide the mechanistic explanation as to how habitat alteration is mediating those changes, effects that are in fact, mediated through changes in the individual's behavioral ecology (Anthony and Blumstein 2000; Caro 2007). By understanding these effects, we may be better able to predict and sometimes mediate such effects and increase our conservation efforts on particular species (Witherington and Martin 2000; Poot et al. 2008).

For aquatic ecosystems, all but a few have been at some point subjected to some sort of habitat alteration, including acid rain, chemical pollution via release of industrial effluents, and agricultural inputs or other climate change-mediated alteration, including temperature and acidification (Garrels et al. 1973; Schindler 1988; Winder and Schindler 2004; Orr et al. 2005). For many species, choosing another more suitable habitat is not an option because of the closed nature of many water bodies or the large scale effects of some factors, such as ocean acidification. Thus, for aquatic species, habitat alteration is likely to affect many aspects of an individual's life, such as the way they interact with conspecifics and heterospecifics, and their ability to access and assess the quality of food or mates. This in turn could have population-wide consequences by affecting the effective size, growth rate, or reproductive skew of a population (Anthony and Blumstein 2000).

The ability of animals to detect signals and information in their environment is influenced by the background level of “noise” in the environment (Endler 1992; Schröder and Hilker 2008; Lonnstedt et al. in press). This pertains not only to auditory information, but to chemical and visual information as well. Visual information, particularly color, is highly sensitive to varying light levels. An individual prey animal may be able to distinguish colors and shapes of predators during the day, but fail to do so at night. Limited visual acuity may have selected for species that specialize at foraging at dawn and dusk when the ability of prey to see is limited. It may also have selected for prey that limit their activity during crepuscular periods, particularly during the full moon (Clarke 1983). In addition to light levels, turbidity is another major factor influencing the transmission of visual information. Increasing turbidity, caused by eutrophication, changes in nearby land use, or other anthropogenic activities, is an increasing environmental concern (Davies-Colley and Smith 2001; Schwartz et al. 2008). Increasing turbidity often leads to substantive community changes through altering trophic dynamics (van de Meutter et al. 2005). Indeed, the outcomes of predator–prey interactions are highly influenced by levels of turbidity (Gregory 1993; Bonner and Wilde 2002; Lehtiniemi et al. 2005; Zamor and Grossman 2007). Prey may use high turbid conditions to hide from predators, but predators may do the same thing. In both cases, the detection and reaction distance of both participants are likely diminished as turbidity increases (Vogel and Beauchamp 1999; Quesenberry et al. 2007). Studies examining the effects of turbidity on predator/prey interactions have rarely, if ever, considered that the actual recognition of predators and non-predators is influenced by turbidity (Ferrari et al. 2010a), but there may be good reasons to believe that this may be the case. In a pioneering study, Seehausen et al. (1997) demonstrated that anthropogenic increases in turbidity interferes with mate choice, relaxes sexual selection, and blocks mechanisms of reproductive isolation in cichlid fish. The aim of our study was to examine whether recognition of predators and non-predators is influenced by turbidity.

The field of behavioral ecology is rife with examples of prey species that show remarkable abilities to adjust when, where, and how they forage and reproduce in response to the presence of predators (Sih 1987; Lima 1998; Stankowich and Blumstein 2005). A fundamental pre-requisite for such anti-predator defenses is the ability of prey to recognize species that pose a threat from those that do not (Ferrari and Chivers 2011). Consequently, it is not surprising that many prey species have a remarkable ability to quickly and efficiently learn the identity of unknown predators (Mathis et al. 1996; Brown and Chivers 2005). In many predator–prey systems representing a variety of taxa, a single conditioning event is enough to facilitate learned recognition of predators (Chivers and Smith 1998; Griffin 2004; Ferrari et al. 2010b). Such efficient learning of predators is somewhat paradoxical. If novel cues can be associated with risk so easily, then there must be frequent opportunities for the prey to inadvertently learn to recognize non-predators as dangerous (Acquistapace et al. 2003; Hazlett 2003; Ferrari and Chivers 2006; Mitchell et al. 2011). Wasting time and energy responding to non-predators is costly; hence, mechanisms should exist to recognize non-predators as safe. Ferrari and Chivers (2011) have argued that understanding how prey animals learn to recognize non-predators and safe places is among the most underappreciated aspect of anti-predator decision making. When we consider this issue, it is important to make a clear distinction between when an animal is not responding to a stimulus because it is not recognized as risky and an animal not responding to a stimulus because it is recognized as non-risky. If an animal is recognized as non-risky, then attempts to condition the prey to associate it with risk may fail. In contrast, if an animal is not recognized as either risky or non-risky, then attempts to condition the prey should be successful (Mitchell et al. 2011).

Latent inhibition and learned irrelevance are mechanisms of learning that can be used by prey to recognize stimuli as non-risky (Acquistapace et al. 2003; Hazlett 2003). Repeated exposure to the unknown stimulus in the absence of risk leads to the stimulus being categorized as non-risky. For example, when virile crayfish, *Orconectes virilis,* have been exposed to the odor of goldfish (*Carrasius auratus*) for 2 h over three consecutive days, they subsequently fail to learn to recognize the cue of the goldfish as a danger (Acquistapace et al. 2003). Likewise, exposing fathead minnows (*Pimephales promelas*) to brook trout (*Salvelinus fontinalis*) odor once a day for 6 days prevented the minnows from learning that the trout was a threat during a one-time conditioning paradigm (Ferrari and Chivers 2006). Mitchell et al. (2011) demonstrated latent inhibition in damselfish (*Pomacentrus moluccenis*), and that latent inhibition could be reversed when the prey were conditioned multiple times. These results illustrate the flexible adjustments that occur with respect to recognizing the level of risk associated with heterospecifics.

An exciting development in predator recognition learning is the finding that prey animals can use the information they have learned about one predator to respond appropriately to other potential predators with which they have no experience. This process is termed generalization of predator recognition. In a landmark study, Griffin et al. (2001) showed that Tammar wallabies (*Macropus euhenii*) that were conditioned to recognize red foxes (*Vulpes vulpes)* as predators were able to generalize this recognition to the sight of cats (*Felis catus*), but not to the sight of goats (*Capra hircus*). Foxes and cats must share some morphological feature, perhaps frontally placed eyes, that allows generalization of predator recognition. Since this finding, several other studies have also demonstrated generalization of not only the sight (Stankowich and Coss 2007; Ferrari et al. 2010a) and odor of predators (Ferrari et al. 2007; Brown et al. 2011) but the relative risk associated with them (Ferrari et al. 2008; Ferrari and Chivers 2009). More recently, two studies have considered whether animals could also generalize their recognition non-predators. Brown et al. (2011) used latent inhibition to teach juvenile rainbow trout (*Oncorhynchus mykiss)* to recognize the odor of pumpkinseeds (*Lepomis gibbosus*) as a non-risky species. Subsequent attempts to condition the trout to recognize the pumpkinseed odor were unsuccessful, as were attempts to condition the trout to recognize novel longear sunfish (*Lepomis megalotis*) odor. The trout that were taught to recognize pumpkinseeds as non-predators had no problem learning novel yellow perch (*Perca flavescens*) as a threat. Perch belong to a different family from the sunfishes. These results convincingly demonstrate fish can generalize recognition of non-predators. Similarly, Ferrari and Chivers (2011) demonstrated that woodfrogs (*Lithobates slyvatica*) taught to recognize salamander (*Ambystoma tigrinum*) odors as non-risky generalize their recognition to closely related newts (*Cynops pyrrhogaster*), but not to goldfish. To date, no studies have tested whether prey animals can generalize recognition of non-predators using visual cues.

In this study, we condition fathead minnows to recognize brook trout as either a predator or a non-predator (through latent inhibition) and then test whether the minnows can generalize their recognition of the trout as a predator or non-predator when they are exposed to morphologically similar looking rainbow trout, but not novel yellow perch. Perch are predatory fish that are novel to the minnows, but are morphologically distinct from either trout species. Immediately following the completion of the first experiment, we repeat the experiment under conditions of increased turbidity to assess whether a reduction in the availability of visual information, as often occurs with anthropogenic disturbance to waterbodies, impairs the ability of fish to learn and generalize recognition of predators and non-predators.

## Methods

### Experimental overview

The experiment consisted of three phases. In stage 1, we exposed minnows to either the sight of a brook trout in an adjacent tank for 1 h twice a day for 3 days, or to the sight of an empty tank. This procedure allows the trout-exposed minnows to learn, through the process of latent inhibition, that the trout do not represent a threat. The positioning of the trout (see below) prevented them from striking at the minnows. In contrast, the minnows that did not experience repeated exposure to the predator in the absence of risk would not have learned the predator as non-risky. In stage 2, we conditioned both groups of fish from stage 1, to recognize one of three different fish as a predator: brook trout, rainbow trout, or yellow perch, by pairing chemical alarm cues with the sight of the predator. In stage 3, we tested each of the groups of minnows to the sight of each of the three fish species. This resulted in 18 treatment combinations in a 2 × 3 × 3 design (2 levels of pre-exposure crossed with three conditioning groups crossed with exposure to three test fish). The experiment was conducted in clear water and was repeated under conditions of reduced visibility by adding bentonite to the tanks containing the predators during the testing phase. Given that generalization of non-predator recognition with visual cues had never been attempted in any predator–prey systems, it was not justified to run the clear and turbid trials at the same time. If the fish failed to exhibit generalization of recognition of the non-predator under clear conditions, then it would have been an excessive waste of animals to conduct all 36 treatments as a single experiment.

### Test species and predators

Minnows used in this study were captured from a pond on the University of Saskatchewan campus in October 2010. Minnows from this pond are naive to all of the predators used in our study and do not exhibit anti-predator behavior in response to them (Ferrari et al. 2010a). Brook trout and rainbow trout were supplied by Reister Trout Farm in Alliance, Alberta, in July 2011, while perch were captured using seine nets in Blackstrap Lake in south-central Saskatchewan in August 2011. Each of the species was maintained in separate 12000-liter tanks in the RJF Smith Center for Aquatic Ecology, until the experiments began. Fish were maintained on a 14:10 h light:dark cycle. Minnows were fed daily with fish flakes (Nutrafin basix, Rolf C. Hagen, Inc., Montreal, Quebec, Canada). Trout were fed daily with commercial trout pellets and the perch were fed twice per week with minnows.

### Stimulus preparation

Fathead minnows, like a variety of other aquatic species, learn to recognize the sight or odor of predators when they detect the cues of the predator in conjunction with chemical alarm cues released by other minnows (Chivers and Smith 1994a; Brown et al. 2001; Ferrari et al. 2010b). This is a highly efficient form of learning that requires a single conditioning event. To prepare the alarm cues (AC), we used 10 donor fathead minnows (fork length, FL: mean ± SD = 5.22 ± 0.48 cm). In accordance with our animals care protocol, the minnows were killed by a blow to the head. We then removed a skin fillet from both sides of each fish, and placed them in chilled distilled water. We collected a total of 23.1 cm^2^ of skin. Skin fillets were homogenized using a Polytron homogenizer, and the solution was strained through glass wool and diluted to obtain a final concentration of 1 cm^2^ of skin per 20 L of water. This concentration has been shown to elicit strong anti-predator responses in minnows (Ferrari et al. 2005).The stimulus was frozen at −20°C in 20-mL aliquots until needed.

### Experimental set-up

The experimental tanks used for the pre-exposure, conditioning, and testing phase were similar. We set pairs of 37-L tanks (50 × 30 × 25 cm) beside each other, such that the long side of the tanks faced each other. The prey's tank contained a gravel substrate, an air stone, a 2-m long plastic stimulus injection tube, as well as a 10 × 20 cm ceramic tile mounted on three 3.5-cm long glass legs. The tile object served as a shelter for the prey. The predator tank was similar except it lacked the injection tube and shelter object, but instead contained Plexiglas dividers that separated the tank into thirds along the long axis of the tank. When the predators were placed in the outer section of the tank (such that minnows in the adjacent tank could see them), they had a restricted ability to move toward or away from the prey. Their alignment in the tank ensured that all of the minnows were presented with comparable visual information across trials (i.e., they had a lateral view of the predator). Each pair of tanks was wrapped on the outer and back sides with plastic to ensure visual isolation from adjacent tanks. A removable divider was used to separate the predator and prey tanks when needed.

#### Pre-exposure phase

Minnows were placed in the prey tanks the day prior to the pre-exposure phase and were fed. The following day we removed the barrier between their tank and the predator tank twice for a 1-h period. This was repeated for 3 days. The predator tanks contained either a single brook trout, or a tank filled with just water. By exposing the minnows repeatedly to trout without any associated risk, the minnows should have the opportunity to learn, through latent inhibition, that trout do not represent a threat. We used 10 different brook trout (fork length, FL: mean ± sd = 20.5 ± 2.4 cm) and randomly assigned them to the tanks during the trials.

#### Conditioning phase

**Figure 1:**
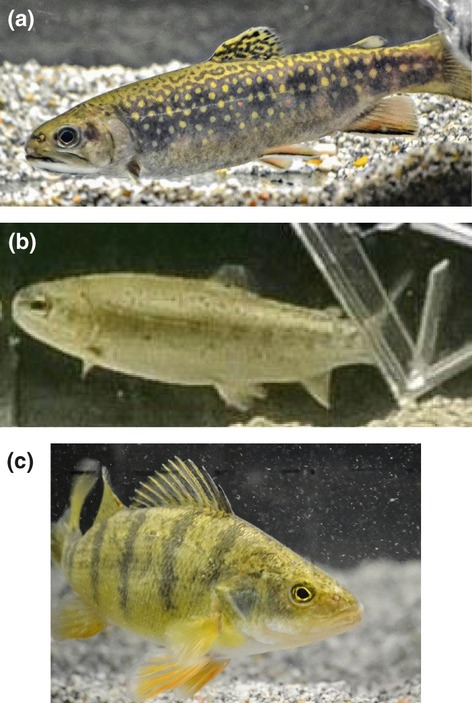
Representative photos of brook trout (panel a), rainbow trout (panel b), and yellow perch (panel c) used as predators and non-predators in the experiments. We used a total of 10 individuals of each species in the experiments. Photos were taken in clear water.

Upon completion of the pre-exposure phase, minnows were placed individually into testing tanks and the conditioning phase was completed the following day. Minnows were fed 2 h prior to the start of the trials. The conditioning stage started when we removed the barrier between the predator and prey tanks and immediately introduced 25 mL of AC in the prey's tank. The AC was flushed into the tank with 60 mL of tank water. The stimulus tanks contained one of three different fish (Fig. 1: brook trout (fork length, FL: mean ± SD = 20.5 ± 2.4 cm), rainbow trout (fork length, FL: mean ± sd = 22.4 ± 2.3 cm), or yellow perch (fork length, FL: mean ± SD = 20.3 ± 2.6 cm). We used 10 individuals of each species of predator and randomly assigned them to the tanks during the trials. After 1 min, we replaced the barrier between the two tanks. We conditioned 120 minnows to each of the three predators, 60 that had been pre-exposed to brook trout and 60 which had not. No behavioral observations were taken during the conditioning phase. Two hours following the completion of the conditioning phase, the minnows were transferred to similar 37-liter tanks containing clean water.

#### Testing phase

We started the testing phase 24 h after the conditioning phase, feeding the fish 1 h prior to the start of the trials. During the testing trials, we recorded the behavior of the minnows for 8 min prior to and 8 min after the removal of the barrier between the tanks. The predator tanks contained brook trout, rainbow trout, or perch. We recorded two well-documented anti-predator responses in single fathead minnows: increased shelter use and decreased activity level (Mathis et al. 1993; Chivers and Smith 1994b, 1995). We measured the number of seconds spent under shelter and the number of seconds the fish spent swimming during both the pre- and post-stimulus periods. There were 20 replicates for each of the 18 treatments in our experiment. Each minnow was used only once. The order of testing was randomized and the observer was blind with respect to the treatments.

### Manipulating turbidity

**Figure 2:**
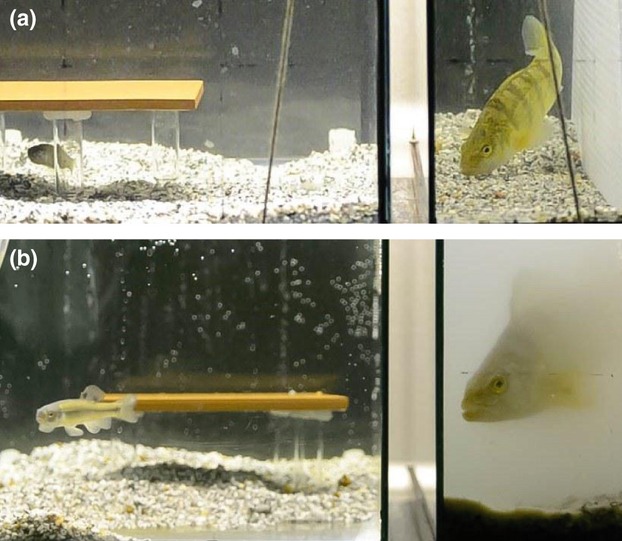
Photos of fathead minnow and yellow perch in the experimental tanks. Yellow perch were photographed in clear water (panel a) and in turbid water (panel b).

Following the completion of the original experiment, we repeated the entire experiment under conditions of reduced visibility during the testing phase (Fig. 2). The minnows used for this experiment were different from those used in the first experiment. To increase the turbidity of the water, we added 4.5 g of bentonite to the predator's tank (0.12 g/L) to give each tank ∼31.5 NTU's of turbidity (∼20-cm Secchi depth (Shoup and Wahl 2009)). The protocol was identical to the one described above.

### Statistical analysis

As the two experiments were performed at different times, the data had to be analyzed separately. We calculated a percent change in behavior from the pre-stimulus baseline ([post-pre]/pre). The data had heterogeneous variances, so we rank-transformed them and performed non-parametric analyses. As shelter use and swimming activity are two correlated behaviors, we analyzed the behaviors simultaneously using a MANOVA approach. We tested the effect of pre-exposure (brook trout or empty tank), conditioning (brook trout, rainbow trout, or yellow perch), and testing cue (brook trout, rainbow trout, or yellow perch) on the behavior of minnows using a three-way non-parametric MANOVA. Interactions were further investigated by performing two-way ANOVAs.

## Results

### Response of minnows to conditioning in clear water

**Figure 3:**
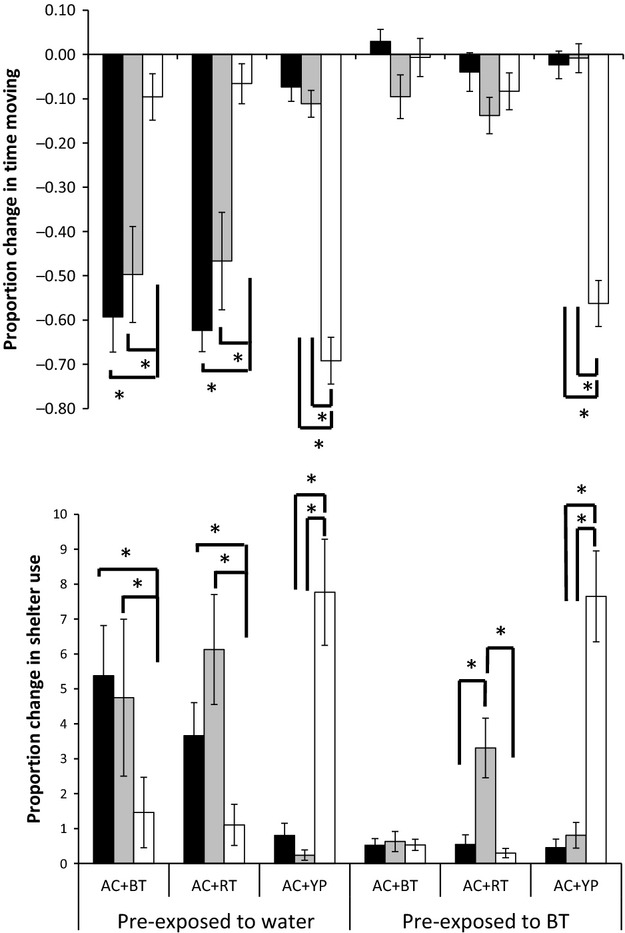
Mean change in proportion in time spent moving (top panel) and shelter use (bottom panel) for minnows exposed to the sight of a brook trout (black bars), rainbow trout (gray bars), or yellow perch (white bars) in clear water. Prior to testing, minnows were pre-exposed to either a tank containing only water (W) or a tank containing a brook trout (BT) and subsequently conditioned with alarm cue (AC) to recognize the sight of a brook trout (AC+BT), rainbow trout (AC+RT), or yellow perch (AC+YP). Asterisks indicate statistical difference at alpha = 0.05, for Tukey post-hoc tests.

The three-way non-parametric MANOVA revealed a significant interaction among pre-exposure, conditioning, and testing cues on the behavior of minnows (Pillai's Trace: H_8,684_=3.0, *P* = 0.002, Fig. 3). To investigate the nature of this interaction, we looked at the effect of pre-exposure and testing cue for each conditioning type. When minnows were conditioned to recognize brook trout or rainbow trout, we found a significant effect of pre-exposure and testing cue on their behavioral response (Pillai's Trace: pre-exposure by testing cue interaction: brook trout conditioning: H_4,228_=6.0, *P* < 0.001, rainbow trout conditioning: H_4,228_=6.8, *P* < 0.001). However, this interaction was not present when they were conditioned to recognize yellow perch (H_4,228_=0.9, *P* = 0.48). Minnows conditioned to recognize perch subsequently responded to the perch, but not to either trout species. This indicates that pre-exposure to brook trout interfered with the type of responses displayed by minnows when they learned to recognize another trout, but not when they learn to recognize a non-salmonid species.

Specifically, minnows pre-exposed to an empty tank and conditioned to recognize a brook or rainbow trout displayed anti-predator responses to both brook trout and rainbow trout, but not toward perch (brook trout conditioning: H_4,114_=7.1, *P* < 0.001; rainbow trout conditioning: H_4,114_=10.4, *P* < 0.001, Tukey post-hoc tests, see figures), thereby demonstrating learning and generalization of predator recognition. However, when minnows were pre-exposed to the sight of a brook trout, minnows conditioned to recognize a brook trout failed to respond to any of the three species (H_4,114_=1.4, P = 0.25), demonstrating learning and generalization of non-predator recognition. Those conditioned to recognize a rainbow trout responded with a weak response to the rainbow trout, but failed to respond to the brook trout or the perch (H_4,114_=5.2, *P* = 0.001, Tukey post-hoc tests, see Fig. 3).

### Minnows exposed to predators in turbid water

**Figure 4:**
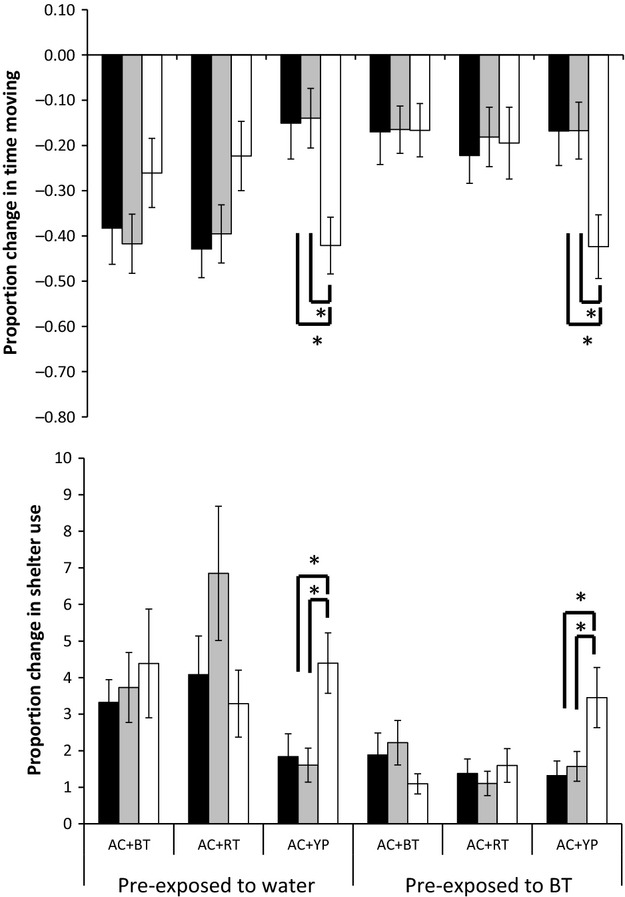
Mean change in proportion in time spent moving (top panel) and shelter use (bottom panel) for minnows exposed to the sight of a brook trout (black bars), rainbow trout (gray bars), or yellow perch (white bars) in turbid water. Prior to testing, minnows were pre-exposed to either a tank containing only water (W) or a tank containing a brook trout (BT), and subsequently conditioned with alarm cues (AC) to recognize the sight of a brook trout (AC+BT), rainbow trout (AC+RT), or yellow perch (AC+YP). The turbidity was manipulated in the testing phase only. Asterisks indicate statistical difference at alpha = 0.05, for Tukey post-hoc tests.

The three-way non-parametric MANOVA revealed a significant effect of pre-exposure (Pillai's Trace: H_2,341_=9.0, *P* < 0.001, Fig. 4), and a significant interaction between conditioning and testing (Pillai's Trace: H_8,684_=3.0, *P* = 0.002), but no other significant main or interactive effects (all *P* > 0.05). When minnows were conditioned to recognize brook trout, their behavioral response was affected by pre-exposure (Pillai's Trace: H_2,113_=7.2, *P* < 0.001), but not by testing cue (H_4,228_=0.7, *P* = 0.57) or any interactive effects between pre-exposure and testing cue (H_4,228_=0.6, *P* = 0.67). This indicates that minnows pre-exposed to brook trout did not show any anti-predator response to any of the fish, but when pre-exposed to an empty tank, they responded indiscriminately to all the species (the two trout and the perch).The same pattern was observed when minnows were conditioned to recognize rainbow trout (pre-exposure: H_2,113_=6.4, *P* < 0.001; testing cue: H_4,228_=0.9, *P* = 0.44; interaction: H_4,228_=0.9, *P* = 0.43). However, when minnows were conditioned to recognize yellow perch, their response was not affected by pre-exposure (H_2,113_=0.2, *P* = 0.79) nor by a pre-exposure by testing cue interaction (H_4,228_=0.2, *P* = 0.95). Their response varied according to the cue they were presented (H_4,228_=4.6, *P* < 0.001). Specifically, minnows displayed an anti-predator response toward the perch, but not toward either of the trout species (Fig. 4).

## Discussion

The results of our studies demonstrate that minnows have the ability to learn to recognize predators through a single conditioning event and then generalize this recognition to the sight of both predators and non-predators. These results confirm the results of Ferrari et al. (2010a) who demonstrated learning and generalization of visual predator cues by minnows. Our work here is the first to document visual generalization of non-predators. More interestingly, the ability of minnows to learn and generalize predators and non-predators was dramatically influenced by turbidity.

Learning allows prey animals with the opportunity to continually update the risk level associated with other species in their environment, and as such, is particularly important in environments where predation risk is highly variable in space and time (Ferrari et al. 2009; Brown et al. 2010). There is a mass of literature showing that prey animals can learn to adjust their behavior in response to the presence of predators (Lima 1998; Wisenden and Chivers 2006), but studies that consider recognition of non-predators are quite rare, being limited to two examples in fish (this study, [Brown et al. 2011] and one in tadpoles [Ferrari and Chivers 2011]). Our failure to consider how prey learn to recognize safe locations and non-predators represents a big gap in our understanding of anti-predator decision making in animals. Learning and generalizing recognition of non-predators may be rarer than learning and generalizing recognition of predators. This stems from the fact that there is a large asymmetry in the cost of making recognition mistakes (Ferrari and Chivers 2011). For learning and generalizing predators, a recognition mistake would mean that the prey would label a novel non-predator as a threat and would waste time and resources responding unnecessarily. However, in the context of learning and generalizing non-predators, a recognition mistake would mean labeling a predator as non-threatening. Such a mistake could lead to death of the prey. In contrast, it is possible that in some ecosystems, a given individual may experience more non-predatory encounters than predatory ones. In such a case, the opportunity for non-predator learning and generalization might be greater. Comparing and contrasting the occurrence of predator versus non-predator, generalization in ecosystems differing in their community composition (ratio of predators-to-non-predators, for instance) may prove fascinating.

There is increasing concern that eutrophication, changes in land use practices, and other anthropogenic activities are leading to substantial increases in turbidity. Turbidity results in benthic smothering, changes rates of photosynthesis, and often results in significant changes in community structure (Bilotta and Brazier 2008; Liljendahl-Nurminen et al. 2008). Predator–prey interactions are often dramatically altered in response to changes in turbidity. Our study provides strong evidence that one of the potential mechanisms of such alterations may be mediated through changes in behavior associated with recognition of predators and non-predators.

Let us first consider the influence of turbidity on learning and generalization of predators. In clear water, fish showed a sophisticated ability to learn and generalize recognition of predators. Fish conditioned to recognize either trout species showed a much stronger response to either of the trout than to the perch, whereas fish taught to recognize the perch showed stronger responses to perch, but not to either trout. In contrast, minnows that were conditioned to recognize either brook trout or rainbow trout in turbid water responded with the same intensity to either trout or perch, indicating that they could not differentiate between trout and perch. Surprisingly, fish conditioned to recognize perch in turbid conditions showed a much stronger response to perch than to either trout species. This seems to indicate that the characteristic(s) that fish use to learn and generalize recognition of trout to perch may be different from what they use to learn about perch. Cues used to distinguish among predators include body shape, as well as differences in color and striped markings on the side of the fish.

Turbidity also had a considerable influence on learned recognition and generalization of non-predators. In both clear and turbid water, when minnows were pre-exposed to brook trout and conditioned to recognize brook trout as a predator, they did not respond to either trout species, indicating generalization of non-predator recognition. However, minnows that learned brook trout as a non-predator could still be conditioned to learn that rainbow trout was a threat in clear water, but could not do so in turbid water.

In addition to the differences in patterns of predator and non-predator recognition that we observed in turbid versus clear conditions, we also had differences in overall intensity of the responses. Looking at both response variables, we find greater intensity responses in the clear conditions than in turbid conditions. This observation fits with Hartman and Abrahams (2000) suggestion that minnows may be less vulnerable to some predators under turbid conditions and hence may reduce their anti-predator behavior accordingly. This result may also be interpreted as a failure of the minnow to perfectly recognize the predator, leading to a truncated response. Both scenarios are supported by the data, but quite difficult to tease apart.

Our study provides strong evidence that turbidity influences anti-predator learning and generalization of predators and non-predators. In our experiment, we tested fish under conditions of approximately 31 NTU's of turbidity, roughly equal to a Secchi depth of approximately 20 cm. This level of turbidity is below the level that minnows may find themselves (Hartman and Abrahams 2000). If anthropogenic activities act to dramatically increase levels of turbidity, then the effects we observed here may be conservative. Indeed, impaired recognition may be even more severe that we documented when turbidity levels really begin to rise. In our experiment, we exposed prey to the predator for a duration of 1 min. This seems like a relatively long time for the prey to have the opportunity to learn the characteristics of the predator. However, it might not represent a long time for species exposed to non-predators, as they may be foraging alongside each other. Changing the duration of exposure could influence learning and generalization abilities. In fact, as turbidity levels increase, prey may need more time to differentiate predators from non-predators.

Individuals are always living “on the edge”, balancing the trade-off between the benefits of successful predator detection and avoidance and those associated with foraging, territorial defense, and reproduction. Their ability to make adequate decisions about which behavioral option to pursue at any point in time (where and how long to forage, which habitat or mate to choose, etc.) is grounded to their ability to collect many reliable pieces of information that inform them about the conditions of their environment. These pieces of information can even be used to assess the likely quality of new food sources or the predatory or non-predatory status of new species (Ghirlanda and Enquist 2003). By changing the quality of their environment, we also change the reliability of the information individuals use in their decision-making process, which means that we are altering the expected pay-off associated with these decisions. Such alterations can lead to decreased foraging gain, reproductive output, or even death, if their vulnerability to predation risk is under-assessed. Thus, from a population perspective, such habitat alterations may excerpt considerable indirect negative effects in population size and growth rate. Whether species can quickly learn to readjust the relationship between information perceived and pay-offs is likely dependent on the type of alterations. Understanding how natural and anthropogenic changes in background levels of “noise” in the environment, be they increases in visual, chemical, or auditory disturbance, will influence this vital process should prove to be fascinating. Conservation biologists and policy makers that make decisions about aquatic resource use are often at a lost to understand mechanisms of change in communities that are associated with what appear to be relatively subtle changes in abiotic conditions. Our study points to alterations in trophic dynamics as potentially a key variable to consider.
